# “Waiting for DAAs”: A retrospective chart review of patients with untreated hepatitis C in Rwanda

**DOI:** 10.1371/journal.pone.0174148

**Published:** 2017-03-21

**Authors:** Neil Gupta, Jules Kabahizi, Constance Mukabatsinda, Timothy David Walker, Emmanuel Musabeyezu, Athanase Kiromera, Jennifer Ilo Van Nuil, Kevin Steiner, Joia Mukherjee, Sabin Nsanzimana, Aimable Mbituyumuremyi

**Affiliations:** 1 Division of Global Health Equity, Brigham & Women’s Hospital, Boston, Massachusetts, United States of America; 2 Partners In Health / Inshuti Mu Buzima, Rwinkwavu, Rwanda; 3 Rwanda Military Hospital, Kanombe, Rwanda; 4 University Teaching Hospital of Kigali, Kigali, Rwanda; 5 University Teaching Hospital of Butare, Butare, Rwanda; 6 King Faisal Hospital, Kigali, Rwanda; 7 Maryland Global Initiatives Corporation, Kigali, Rwanda; 8 Rwanda Biomedical Center, Kigali, Rwanda; University of Cincinnati College of Medicine, UNITED STATES

## Abstract

**Background:**

Access to treatment for hepatitis C virus (HCV) in sub-Saharan Africa is extremely limited. With the advent of direct acting antivirals (DAAs), highly effective and easy-to-deliver oral regimens are now available on the global market. This study was conducted to understand the background and characteristics of a national cohort of patients with HCV infection enrolled in care and awaiting therapy with DAAs.

**Methods and findings:**

We conducted a retrospective chart review of all adult patients with confirmed HCV infection who were currently enrolled in care and treatment at the four existing hepatitis referral centers in Rwanda. Patient charts at these centers were reviewed, and routinely collected data were recorded and analyzed. Overall, 253 patients were identified; median age was 56 years (IQR: 43, 65), and 149 (58.9%) were female. Median viral load was 688,736 IU/ml and 96.7% were HCV genotype 4. As classified by FIB-4 score, 64.8% of the patients had moderate to severe fibrosis. Fibrosis stage was associated with age (OR 1.12, CI 1.09–1.17), but not with time since diagnosis, gender, treatment center, or type of insurance. There was a low frequency of documented co-morbid conditions, including hypertension, diabetes, HIV, and hepatitis B virus.

**Conclusions:**

Compared to an estimated 55,000 patients eligible for HCV treatment in Rwanda, this study identified only 253 patients currently diagnosed and engaged in care, highlighting an immense treatment gap in HCV, likely due to the lack of accessible and affordable screening, diagnostic, and treatment modalities. The patients that were enrolled in care had a disproportionately advanced fibrosis stage, possibly indicating late presentation to care or lack of treatment options. In the context of newly available and effective treatment options, this study supports the overall need to accelerate access to HCV screening, diagnostics, and care and treatment services in resource-limited settings in sub-Saharan Africa.

## Introduction

Hepatitis C virus (HCV) infection is a global pandemic affecting an estimated 115–185 million individuals, more than 80% of whom remain chronically infected, and accounts for between 300,000 and 700,000 deaths annually [1–3]. Approximately 20% of these infections are estimated to occur in sub-Saharan Africa (SSA) [4], and this accounts for the second greatest number of infected individuals after the Eastern European region [5]. Seroprevalence for HCV in SSA has been reported to be 2.7% overall, ranging from 0.7% in southern and eastern Africa to 6.9% in central Africa [6]. In Rwanda, a country of 11.3 million people in the Great Lakes region of Africa [7], HCV seroprevalence has been estimated between 3.1–4.1% via antenatal care, blood donor and HIV screening programs [8–10]. Co-infection with HIV and HCV is also a public health concern in SSA, and the prevalence of HIV-HCV co-infection in SSA has been reported to be 5.7%, with significant regional variation [6]. In Rwanda, the prevalence of HCV in people living with HIV is estimated between 4.9–5.7% [10, 11].

Prior to 2012, the treatment of HCV required interferon. This expensive and complex therapy was beyond the reach of most SSA countries to provide at scale. In addition, diagnostic tools to diagnose and stage HCV are not widely available. Because of the limited diagnostic and treatment options for HCV in SSA, there are no cohort studies describing the risk factors, clinical presentation or socio-economic characteristics of patients with HCV. Since 2012, a number of all-oral regimens were shown to be curative [12], and the simplicity of these curative regimens has sparked discussion for treatment equity across the globe [13, 14]. In preparation for the implementation of oral, direct acting antiviral (DAA) treatment regimens, patients are now being identified to receive treatment in Rwanda. This study describes patients diagnosed and enrolled in care for HCV currently awaiting oral curative treatment in Rwanda.

## Methods

### Setting

Rwanda is well positioned to implement expanded screening and treatment of HCV due to strong leadership and extensive investment in health care infrastructure over the past two decades, driven largely by the response to the HIV epidemic [15]. As of 2015, Rwanda reported 81.5% treatment coverage for HIV, with 153,147 individuals currently on antiretroviral therapy and in care and treatment programs. Five-hundred twenty-four health facilities now offer HIV care and treatment, and initiation and follow-up of first-line therapy for adults and children has been shifted to the primary care level [16]. The public community-based health insurance scheme provides health insurance to 80.9% of the country’s population [17], requiring a minimal annual premium and 10% co-insurance for facility visits, diagnostics, and medications. The remaining insured population has health insurance provided by public insurance available to civil servants (2.7%), active or former military (0.3%), and private sector (0.4%) [18].

As of 2016, antibody testing for HCV in Rwanda was available at 13 facilities using ELISA and at least two private diagnostic facilities. Serologic testing for HCV is paid for by most private insurance plans and by the public national insurance scheme with a co-pay of ~$5 USD per test. HCV quantitative PCR testing is available at two referral laboratories at a cost of ~$100 USD per test, and genotyping at one referral center at a cost of $133 USD; a variable portion of these tests are paid for by both private insurance and public national insurance schemes [19] Before 2015, interferon combined with ribavirin was the sole approved treatment regimen for HCV in Rwanda. Because of the expense and complexity of the regimen (estimated at approximately $10,500 USD per regimen), it was available only at the four referral hospitals and administered by specialized physicians. As of January 2015, 92 patients were reported to have received treatment in Rwanda with interferon/ribavirin therapy, of whom 24 achieved sustained virologic response and 13 discontinued therapy prior to completion (unpublished data). In 2011, the Rwanda Ministry of Health established a technical working group on viral hepatitis, which included members from the Rwanda Ministry of Health, referral hospital physicians, university faculty, implementing partners, and private industry. This group produced the first national guidelines for viral hepatitis diagnosis and treatment, which were adopted in 2013 and addressed both hepatitis B and C. The guidelines were updated in 2015 to include additional treatment options of sofosbuvir/ribavirin, sofosbuvir/ledipasvir, and sofosbuvir/daclatasvir and the working group developed the National Operational Plan for Hepatitis in 2016 [20].

### Study design, data collection, data analysis

We conducted a retrospective chart review of all adult patients >18 years old with known, untreated HCV infection followed at four referral treatment centers in Rwanda: University Teaching Hospital of Kigali, University Teaching Hospital of Butare, Rwanda Military Hospital, and King Faisal Hospital. Patients were required to have previously documented HCV infection confirmation by HCV PCR quantification. We extracted de-identified sociodemographic and clinical data on all patients from routinely collected information contained in patient files from June-August 2015. The presence of documented co-morbidities, including HIV, hepatitis B virus (HBV), tuberculosis, hypertension, diabetes, depression, and cancer were recorded. Associations between categorical variables were determined using Chi-squared analysis and Fisher’s Exact Test, and associations between categorical and continuous variables were assessed using logistic regression (STATA v13.1). FIB-4 score was calculated by the following formula: FIB4 = [age (years) x AST(IU/L)]/[platelet count(10^9^/L) x ALT(IU/L)^1/2^]. FIB4 results were categorized according to validated low and high cutoff values corresponding with significant fibrosis (METAVIR ≥2) [21]. Ethical approval was obtained from Rwanda National Ethics Committee and Partners Institutional Review Board for this study.

## Results

Overall, 253 patients were registered in follow-up for active HCV infection and awaiting treatment at the four referral centers. Of these patients, median age was 56 years (IQR: 43, 65), and 149 (58.9%) were female (Table 1). Of those with insurance data (n = 110), 44 (40.0%) had public servant insurance, 35 (31.8%) were covered by the national health insurance scheme, and 31 (28.2%) had private insurance.

**Table 1:** Baseline demographics of Rwanda hepatitis C registry patients (N = 253).

Characteristic	N	n(%)
Gender	253	
Female		149 (58.9)
Male		104 (41.1)
Age group (years)	238	
18–29		6 (2.5)
30–39		38 (16.0)
40–49		41 (17.2)
50–59		61 (25.6)
60–69		68 (28.6)
>70		24 (10.1)
Insurance Status	110	
CBHI*		35 (31.8)
Public Servant		44 (40.0)
Private		31 (28.2)

N = total number of patients with data available for analysis

n = total number of patients within each sub-group

*Community Based Health Insurance

Hypertension was the most commonly documented comorbidity (n = 17), followed by diabetes (n = 10), and HIV (n = 10) (Table 2). Seven patients had documented co-infection with hepatitis B. Twenty (7.9%) patients had documented previous HCV treatment, all of whom were treated with interferon and ribavirin; 17 (85.0%) of these patients completed treatment without cure, 2 (10.0%) did not complete treatment, and 1 (5.0%) had unknown treatment completion status. Previous treatment was associated with receiving care at the private referral hospital, King Faisal Hospital (p = 0.003, Fisher’s exact test), but not with gender (Χ^2^ 0.01, p = 0.92) or with having private insurance (p = 1.00, Fisher’s exact test).

**Table 2:** HCV history characteristics of Rwanda hepatitis C registry patients who have not been cured as of July 2015 (N = 253).

Characteristic	N	n(%)
Time since initial diagnosis	225	
0–6 months		32 (14.2)
6–12 months		35 (15.6)
1–5 years		139 (61.8)
5–10 years		14 (6.2)
>10 years		5 (2.2)
Facility where diagnosed	220	
Referral hospital		213 (96.8)
District hospital		5 (2.3)
Other		2 (0.9)
Previous HCV treatment	253	
None		253 (100)
RIbivirin + interferon		0
Oral direct acting antiviral agent		0
Frequency of documented co-morbidities		
HIV on ART		9
HIV not on ART		1
Hepatitis B		7
Tuberculosis		0
Hypertension		17
Diabetes		10
Depression		0
Cancer		1

N = total number of patients with data available for analysis

n = total number of patients within each sub-group

HCV = hepatitis C virus

ART = antiretroviral therapy

Median viral load was 688,736 IU/ml and 112 (44.3%) had viral load greater than 800,000 IU/ml (Table 3). Of 60 patients with genotype results, 58 (96.7%) were type 4, 1 (1.7%) was genotype 2, and 1 (1.7%) was genotype 3. Of 173 patients with data available for FIB-4 calculation, median FIB-4 score was 1.74 (IQR: 1.16, 3.01) with 61 (35.3%) classified as mild fibrosis, 75 (43.4%) moderate, and 37 (21.4%) severe. FIB4 score was associated with age (OR 1.12 [1.09, 1.17]), but not with time since diagnosis (OR 1.00 [0.99, 1.02]), gender (Χ^2^ 1.25, p = 0.54), treatment center (p = 0.77, Fisher’s exact test), or type of insurance (p = 0.24, Fisher’s exact test). Absence of FIB4 score was not associated with patient age (p = 0.69, Fisher’s exact test) or time since diagnosis (p = 0.42, Fisher’s exact test).

**Table 3:** Clinical and laboratory parameters of Rwanda hepatitis C registry patients (N = 253).

Laboratory Studies	N	n (%)
Median HCV viral load [log10 IU/ml] (IQR)	253	5.8 (5.4, 6.3)
HCV viral load > 800,000 copies (Iu/ml)	253	112 (44.3)
HCV genotype	60	
1		0 (0)
2		1 (1.7)
3		1 (1.7)
4		58 (96.7)
Albumin < 3.5	166	19 (11.4)
Platelets < 90,000/mm^3^	187	9 (4.8)
FIB-4	173	
≤1.45		61 (35.3)
>1.45 to ≤3.25		75 (43.4)
>3.25		37 (21.4)

N = total number of patients with data available for analysis

n = total number of patients within each sub-group

HCV = hepatitis C virus

## Discussion

This study is the first to our knowledge to describe characteristics of a cohort of patients with HCV in SSA representing a national sample of those enrolled in care and awaiting therapy with DAAs as determined by a retrospective chart review method. Previous studies from this region have focused on estimating the prevalence of HCV infection, primarily through blood donor, antenatal, and HIV screening programs, as well as the epidemiology and mode of infection [22]. Given the public-private nature of the HCV treatment centers in Rwanda and lack of other treatment centers within the country, this cohort likely captures most, if not all, patients that were engaged in care within Rwanda for HCV infection at the time of the study.

In this study, only 253 patients were registered in care with confirmed HCV infection in Rwanda; however, it has been estimated that over 55,000 patients would be eligible for treatment under full coverage of HCV screening, diagnosis, and treatment, highlighting the dramatic gap in the availability and accessibility of diagnosis and care [23]. Notably, a disproportionate number of patients in this cohort had private health insurance compared to the overall population, which likely represents lack of access to HCV diagnosis and treatment through the public sector. Additionally, this may also represent a lack of awareness of hepatitis infection by the general public, which has not been previously emphasized in national public health campaigns, and data regarding hepatitis has not been collected by demographic health surveillance systems or other national health surveys.

It is notable that among patients for whom a FIB-4 score could be calculated, almost two-thirds of this cohort had moderate to severe fibrosis. This may represent a bias for the diagnosis and linkage to care of patients expressing more advanced clinical signs and symptoms, though it may also indicate delays in diagnosis and access to care. While not unexpected, given the lack of availability of wide spread screening and of curative therapy, it demonstrates the need for the expedited expansion of access to curative drugs. Of note, only 7.9% of the patients in this cohort had accessed previous treatment for HCV with interferon and ribavirin.

Overall, there was a low frequency of documented co-morbidities among patients in this cohort. The frequency of documented co-morbid hypertension and diabetes among this cohort are similar to previous rates of hypertension and diabetes reported in the general population in Rwanda [24]. The lack of documented depression may signify the absence of routine screening with validated tools in this population as the depression rate has previously been reported at 15.5–19% in the general Rwandan adult population [24–26]. The low frequency of documented cancer diagnoses may reflect the fact that patients with diagnosed cancer were not referred or registered for treatment at the referral centers due to treatment contra-indication, or it could also suggest lack of systematic screening or underdiagnosis of both hepatocellular carcinoma and other malignancies overall. The frequency of HIV co-infection was slightly lower than expected from aggregated rates of HIV-HCV co-infection in cohorts of HIV infected adults reported across SSA [5], but may simply reflect the background HIV prevalence of the Rwandan population [27]. The frequency of documented HBV co-infection was lower than expected in this cohort compared with global co-infection prevalence estimates of between 2–20%; however, similarly low HBV seroprevalence among high-risk groups in Rwanda have been previously reported [28, 29].

This study was limited by missing data, likely due to the non-standardized documentation resulting in incomplete data. As national screening, monitoring, and management guidelines for HCV have only recently been established, not all parameters that would provide a more thorough assessment of these patients were systematically collected or completely available, likely resulting in underestimation of co-morbidities, including HBV co-infection. Additionally, there was little documentation regarding laboratory, radiographic, and pathology results, and social and epidemiologic risk factors were not systematically recorded nor reviewed in this study. This emphasizes the need for standardized patient charts and registers to collect uniform data to both support individual-level clinical decision making as well as population level monitoring, evaluation, and quality control of the national hepatitis program. It also highlights the need for prospective research studies to more accurately assess the epidemiology, risk factors, clinical stage, and treatment outcomes for patients with HCV in Rwanda [28]. Finally, although a recommended low-cost tool for clinically estimating fibrosis progression in resource poor-settings, the FIB4 scoring method has not been validated for the research setting in populations with the potential for thrombocytopenia due to non-HCV related causes, and it could therefore potentially overestimate the level of fibrosis in this population.

To our knowledge, the current study is the first in this region to provide data on clinical parameters of patients enrolled in care for HCV infection and awaiting treatment. These findings provide support to the imperative for further investigation, capacity building, and improved access to and affordability of DAAs in sub-Saharan Africa. With the advent of DAAs on the global market, referral centers in Rwanda have discontinued interferon-based therapy and are recommending for patients to pursue non-interferon based DAA treatment options. Since the time of data collection for this study, DAAs have become increasingly available and affordable for patients in Rwanda, and a number of patients are currently accessing treatment via preferred pricing offers (approximately $300-$400 USD per month) by licensed manufacturers in addition to increasing coverage by insurance providers. However, even at this reduced price, treatment still remains out of reach for the vast majority of the population. Given this clear need, the Rwanda public health system has developed a national response to HCV, including scale up of rapid diagnostic testing at the district and provincial hospital level, as well as plans for increased capacity for quantitative HCV viral load and genotyping at seven referral hospitals and four provincial hospitals around the country. Existing infrastructure that has supported an effective HIV response in Rwanda, including trained personnel, laboratory capacity, commodity supply chains, and information systems, may serve as a suitable platform for HCV treatment scale-up and decentralization. A central registry has been established to document and record patients identified in care, and standardized hepatitis-specific registers and patient charts have been developed and introduced to guide patient-level medical decision making, standardization of therapy, and monitoring and evaluation of patient outcomes. Now, with the advent of highly effective and increasingly accessible treatment options, there is need for a thoughtful rollout of an equitable public health approach to this previously undertreated epidemic.

## Supporting information

S1 File (DTA)
